# Grazing during the grassland greenup period promotes plant species richness in alpine grassland in winter pastures

**DOI:** 10.3389/fpls.2022.973662

**Published:** 2022-08-16

**Authors:** Wanrong Wei, Qiaoyan Zhen, Jia Deng, Hanlin Yue, Mingsen Qin, Maria K. Oosthuizen

**Affiliations:** ^1^Key Laboratory of Southwest China Wildlife Resources Conservation, College of Life Sciences, China West Normal University, Nanchong, China; ^2^China West Normal University, Nanchong, China; ^3^Department of Zoology and Entomology, University of Pretoria, Hatfield, South Africa; ^4^Mammal Research Institute, University of Pretoria, Hatfield, South Africa

**Keywords:** grassland, grazing, winter pasture, grassland greenup period, plant richness

## Abstract

Although grazing is the most common use of grassland, the ecological function of grassland far exceeds its productivity. Therefore, the protection of plant diversity is of the utmost importance and cannot be ignored. Existing research on the effect of grazing on grassland mainly focuses on grazing intensity and the type of livestock, but the consequences of the timing of the grazing on the vegetation community remains unclear. We investigated plant community characteristics of winter pastures in alpine meadow with different grazing termination times (grazing before and during the grassland greenup periods) in Maqu County, eastern QTP. The results showed that vegetation height, coverage, aboveground biomass and Graminoid biomass were lower in grassland when grazing happened during the greenup period compared to grassland where grazing was terminated before the greenup period. However, the total plant species richness and forbs richness were higher in grassland with grazing during the greenup period compared to grassland without grazing during the greenup period. Our structural equation modeling reveals a potential indirect implication for the total plant species richness and forbs richness of winter pastures mainly through a decrease in the vegetation coverage and grass biomass abundance. Our findings imply that grazing during the grassland greenup period may facilitate the maintenance of plant diversity in winter pastures. These findings have important implications for grassland ecosystem functioning and for the conservation of plant diversity.

## Introduction

Grassland is one of the most abundant type of vegetation in the world, and accounts for nearly 20% of the global land surface (Scurlock and Hall, 1998). The primary function of grassland is productivity, and includes animal husbandry, soil and water protection, nutrient transformation, climate regulation, biodiversity and genetic resource protection (Harris, 2010). Grazing by herbivores is the most common use of the grasslands worldwide (Herrero and Thornton, 2013). Livestock affects grassland vegetation and soil by feeding, trampling, and returning manure and urine (McSherry and Ritchie, 2013). Appropriately managed grazing not only helps to stimulate compensatory growth of plants and accelerates nutrient cycling, but also affects the physical structure of soil and promotes root development, which indirectly affects grassland productivity and plant diversity (Zhang et al., 2018). Thus far, existing research on the consequences of grazing on grassland mainly focuses on grazing intensity and the type of livestock (Bai et al., 2007; Herrero and Thornton, 2013; Li et al., 2019; Zhan et al., 2020). However, the magnitude and direction of the changes that herbivores exert on plant communities are variable and influenced by the grazing time (Vermeire et al., 2008; Zhu et al., 2008). Nevertheless, the relationship between grazing timing and the vegetation on alpine grasslands, in particular how the grazing time relates to plant growth, has been overlooked.

The ecological functioning of grassland far exceeds its productivity. The protection of biodiversity is an important component of grassland ecological function (Zhang and Zhao, 2017). In small-scale family pastures, grassland vegetation characteristics are primarily affected by the grazing intensity and the species of livestock that are maintained and is determined by the differential use of plant species by livestock, and the resistance and limit of plants to livestock feeding and trampling (Li et al., 2019). In addition, the impact of grazing on vegetation communities is also closely related to the stage of plant growth (Wanyama et al., 2020; Miao et al., 2021), and is usually more pronounced in the early stages of plant growth (Nie and Zollinger, 2012; Ma et al., 2017). For example, grazing during the greenup period of grassland may significantly reduce the vegetation height, coverage, aboveground biomass and graminoids biomass (Zhao et al., 2003; Zhu et al., 2008; Ma et al., 2017), and increase plant richness of the degraded grassland in summer pastures (Li, 2018). However, the above-mentioned studies mainly focus on the productivity related indicators of degraded grasslands in summer pastures, while plant diversity has not been extensively studied, which is not conducive to the sustainable development of grassland ecosystems. Plant species richness is the most basic indicator of plant diversity (Tilman et al., 2001), and plays an important role in grassland ecosystem function. Many studies indicated that plant richness can positively increase the function of grassland ecosystems (Tilman et al., 2006; Wanyama et al., 2020; Miao et al., 2021). Therefore, it is necessary to study the effect of grazing during the grassland greenup period on plant richness.

Alpine meadow covers the southeastern quarter of the Tibetan Highlands and form the world’s largest alpine pastoral ecosystem. The grassland contracting system was implemented in the 1980’s in this region (Abuman and Chang, 2012), and since then, the grassland grazing management system changed from the traditional nomadic grazing to sedentary grazing (Abuman and Chang, 2012; He and Kietch, 2019). The most common method of grazing management is nomadic, where livestock moves seasonally between fixed summer and winter pastures (Wei et al., 2020a,b). The livestock species, grazing intensity and grazing time are determined by the individual herdsmen. The grassland greenup period is the period when the plants start to turn green again, and it is a critical period in the lifecycle of plants (Ma et al., 2017). The grazing during this period is an important factor that must be considered in grassland utilization and grassland management. Several studies have investigated the difference in the vegetation community when livestock is allowed to graze during the grassland greenup period as opposed to when grazing is terminated before the grassland greenup period, but only in summer pastures (Zhao et al., 2003; Zhu et al., 2008; Ma et al., 2017). To date, the difference in grassland plant communities when grazing takes place during the grassland greenup period or is terminated before the greenup period in winter pastures has not been investigated.

To narrow this knowledge gap, we conducted a study to explore changes in the plant community resulting from grazing during the grassland greening period in winter pastures at the scale of family pastures. We hypothesized that the total plant species richness will be higher in grassland with grazing during the greenup period in winter pastures because of the higher forbs richness. Firstly, forbs represent the bulk of the plant species in alpine meadows, and secondly, the lower biomass% of the dominant functional groups and vegetation cover facilitates the utilization of light by the understory forbs species. The results of this study are of great significance for the conservation of plant diversity at the scale of family pastures in alpine meadows on the Qinghai–Tibetan Plateau (QTP).

## Materials and methods

### Study site

**Figure 1:**
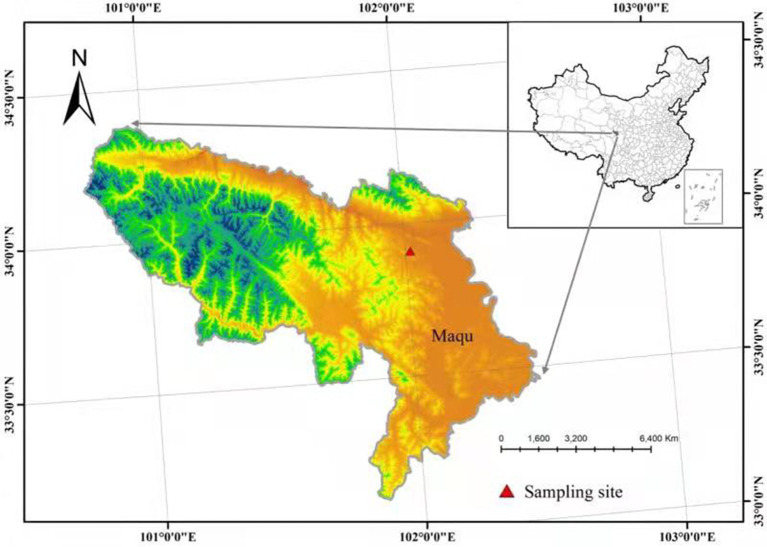
Locations of the sites on the Qinghai–Tibetan plateau.

Our study site is situated at the Hequ racecourse, in Maqu County, eastern QTP in the Gansu Province County, northwestern China (Figure 1). The local climate is typical for an alpine continental habitat. The average altitude is 3,430 m, the mean annual temperature is −2.3°C, and the average annual rainfall is 643.9 mm, and falls predominantly during summer (June to September). The soil type is specific to alpine meadows (Wei et al., 2020b). The vegetation in this area is dominated by Poaceae, Cyperaceae, Ranunculaceae, and Compositae, such as *Kobresia pygmaea*, *Elymus nutans griseb*, *Cremanthodium lineare*, *Anemone rivularis*, *Leontopium leontopodioides*, and *Anemone rivularis*.

### Herdsmen’s winter pasture selection design

This study was carried out between April and August of 2021. We conducted a detailed survey (start of grazing time, end of grazing time, grassland utilization) of one village at the Hequ racecourse between April and May 2021. We screened a total of 9 herdsmen households that meet our requirements which had same grazing start time (November 5), but the grazing end time is differed (4.12–5.13) in their winter pastures. The distance between the selected herdsmen was between 3 and 5 km, and the winter pastures of these 9 herdsmen were randomly distributed. The entire area of the Hequ racecourse was nomadic before the 1980s. After the implementation of the household responsibility system in 1980, winter pastures and summer pastures were grazed in rotation. The transition time was concentrated after the grassland greenup period, but the exact date varied between years. Following the implementation of the livestock reduction policy in 2011, the herdsmen adopted a fixed-time grazing regime. Our survey indicates that the grazing start time and grazing end time of their winter pastures are now essentially the same every year. The transition time of livestock depends on when sufficient plant growth has taken place in the summer pastures to sustain the livestock. The soil type in Hequ racecourse is an alpine meadow soil with a high organic matter content, thick humus stratum, a good soil aggregate structure and sufficient moisture (Wei et al., 2019). Our survey results show that four of the herdsmen moved their livestock from winter pasture to summer pasture before the grassland greenup period, while five herdsmen only moved their livestock from winter pasture to summer pastures during the grassland greenup period. We found that the greenup period of grassland of Hequ racecourse starts on April 24, and ends on May 15 in 2021, according to the technical guidelines for ground observation in China’s grassland greenup period. The ground observation standard of grassland greenup is as follows: when 40%–60% of plants in a projected area turns green, it classified as greenup period. The standard of the quadrats in the grassland greenup period is 1 m × 1 m. The vegetation survey was conducted in early August 2021. This study focused on the impact of grazing on the vegetation community during the grassland greenup period of the winter pasture. The vegetation community survey excluded the impact of small herbivores on grassland vegetation, such as plateau pika (*Ochotona curzoniae*), plateau zokor (*Eospalax fontanierii*), and Himalayan Marmot (*Marmota himalayana*). Therefore, no small herbivore activity is mentioned above in the plot setting area.

### Plant sampling

Vegetation was randomly sampled from 10 quadrats (50 cm × 50 cm) in each winter pasture of a selected herder. The distance between the quadrats were greater than 30 m and the quadrats were set up in areas where there was no small herbivore activity. Vegetation cover and height, species richness, aboveground biomass, and three functional groups biomass, richness, and the Graminoid biomass were determined. Vegetation cover was calculated with the acupuncture method (Wei et al., 2019). Vegetation height was measured by averaging the heights of 30 randomly selected individual plants. Species richness was determined by the number of the species recorded in the 0.25m^2^ quadrats (Wei et al., 2020c). Aboveground community biomass was assessed by clipping all plants at ground level in a 0.25m^2^ quadrat, sorted into forbs, sedges and grasses, drying them at 80°C for 24 h, and weighing them. Functional group richness is the number of species contained in that functional group in a 0.25-m^2^ quadrat. Grasses and Sedges are the preferred forage of livestock, therefore we calculated the Graminoid biomass as the combination of grasses and sedges (Wei et al., 2019). In addition, we calculated the biomass proportion of each functional group according to the following formula: Functional group biomass % = (Functional group biomass/Total biomass) × 100%.

### Statistical analyses

All data were analyzed using SPSS 19.0 for Windows. We checked the data for normality and homogeneity (Shapiro–Wilk test) of variance before analysis. All data were expressed as mean ± standard error (SE). Differences between means were considered significant when the *p*-value of the independent sample t-test was less than 0.05.

Furthermore, we constructed a series of piecewise structural equation models (piecewise SEMs; Lefcheck, 2016) to assess the potential changes in the total plant species richness and forbs richness following grazing during the grassland greenup period in winter pastures. Previous studies have shown that in alpine meadows, plant species richness is not only related to community height and coverage (Hautier et al., 2009; Borer et al., 2014), but also closely related to the dominant functional group biomass % (Liu et al., 2015; Li et al., 2016). In this study, the dominant functional group in winter pastures grazing before regrowth (control) was the grass functional group, so the grass functional group biomass % was also used as one of the variables. The Pearson correlation showed that there was no significant correlation among the vegetation height, coverage and the grass functional biomass% (Supplementary Table S1). Therefore, we hypothesized that grazing during the grassland greenup period would directly impact vegetation cover, vegetation height and the grass functional biomass%, and therefore indirectly influence total plant species richness and forbs richness (Supplementary Figure S1). A step-wise fitting procedure was used to achieve the best-supported model based on the metamodel (Yao et al., 2020). The chi-square test (χ^2^) was performed to test the overall goodness of fit for SEM. It was considered acceptable when the model fit index of χ^2^/df is within the range 0.00–2.00 and *p >* 0.05, RMSEA values with *p <* 0.05 were considered significant (Hu and Bentler, 1999), and non-significant paths (arrows) were deleted in the final model. All statistical analyses were performed using SPSS 19, with significance levels set to *p* < 0.05. All the SEM analyses were conducted using the software Amos 20 (IBM SPSS Inc., Chicago, IL, United States).

## Results

### Grassland plant community with and without grazing during greenup period

**Figure 2:**
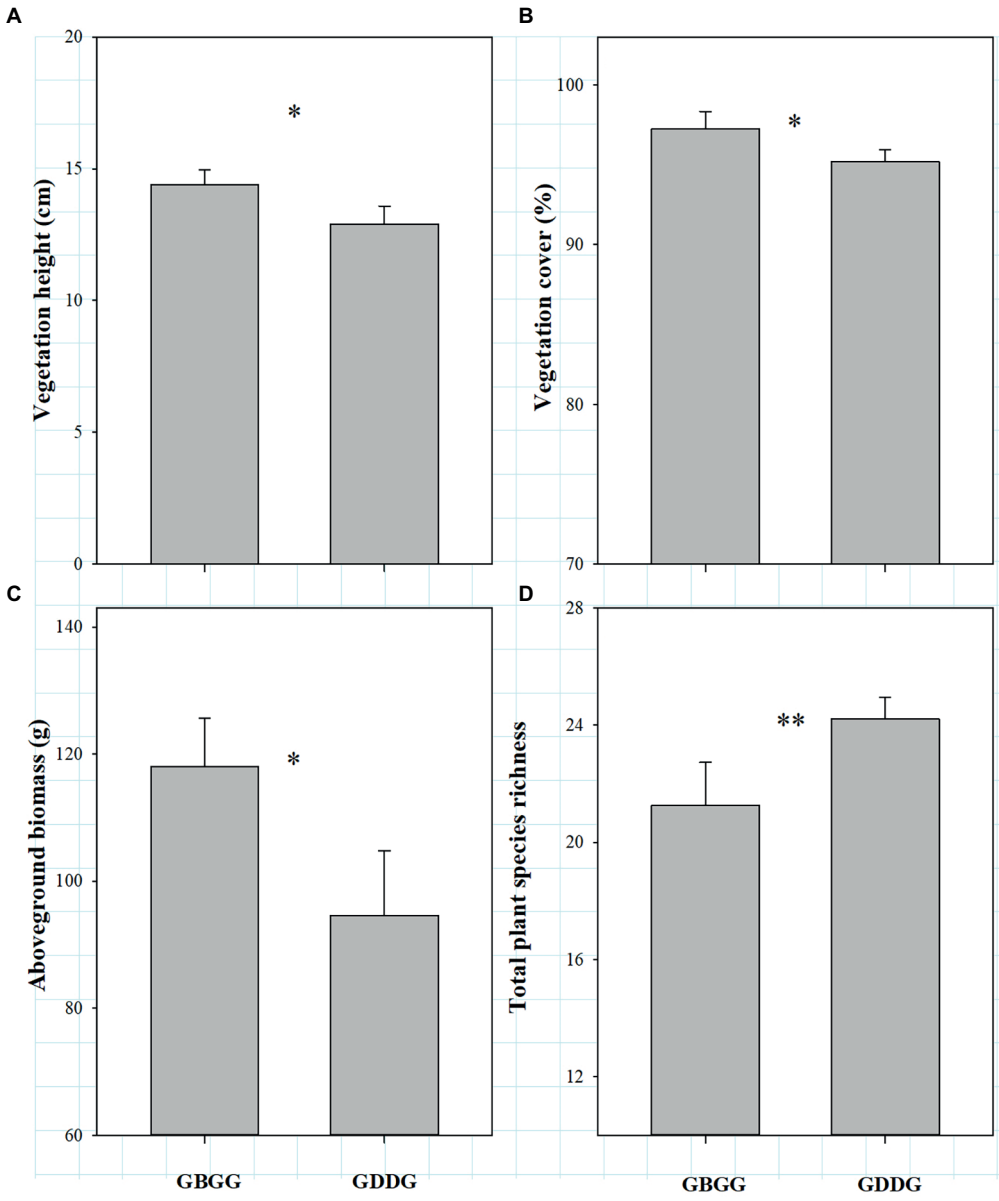
The difference in the vegetation community with or without grazing during the grassland greenup period in winter pastures, **(A)** Vegetation height; **(B)** Vegetation cover; **(C)** Aboveground biomass; **(D)** Total plant species richness. GBGG: grazing terminated before grassland greenup; GDGG: grazing during grassland greenup. * and ** indicate significant difference at 0.05 and 0.01 levels, respectively, ns indicates no significant difference at 0.05 levels.

In winter pastures, the community composition and structure differed between grazing during and before grassland greenup period. Vegetation height, cover and aboveground biomass were lower and total plant species richness was higher when grazing happened during the greenup period compared to when grazing happened before the greenup period (Figures 2A–D).

### Grassland plant functional groups with and without grazing during greenup period

**Figure 3:**
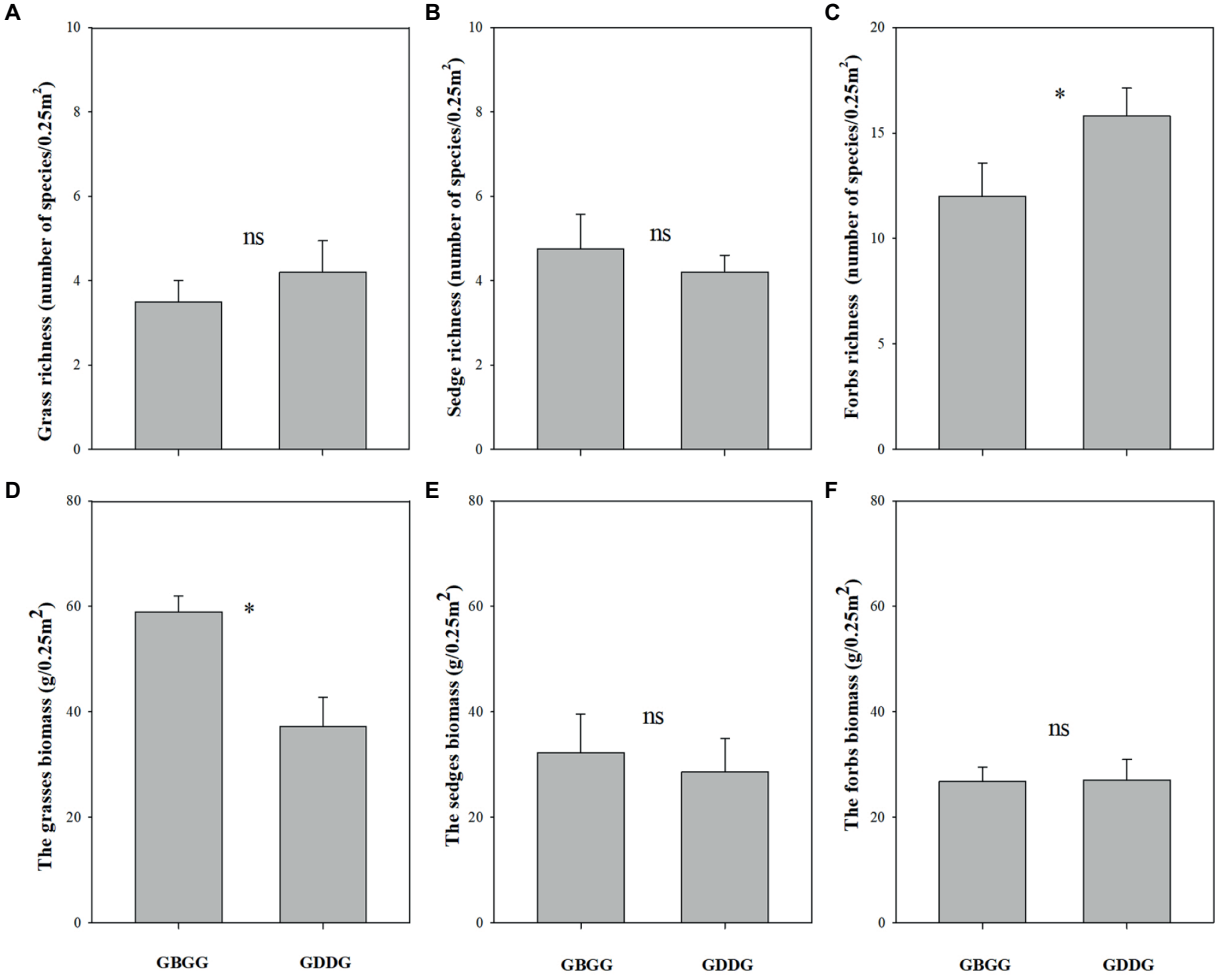
The difference in functional group species richness and biomass with or without grazing during the grassland greenup period in winter pastures, **(A)** Grass richness; **(B)** Sedge richness; **(C)** Forbs richness; **(D)** The grasses biomass; **(E)** The sedges biomass; **(F)** The forbs biomass. GBGG: grazing terminated before grassland greenup; GDGG: grazing during grassland greenup. * indicates significant difference at 0.05 and ns indicates no significant difference at 0.05 levels.

**Figure 4:**
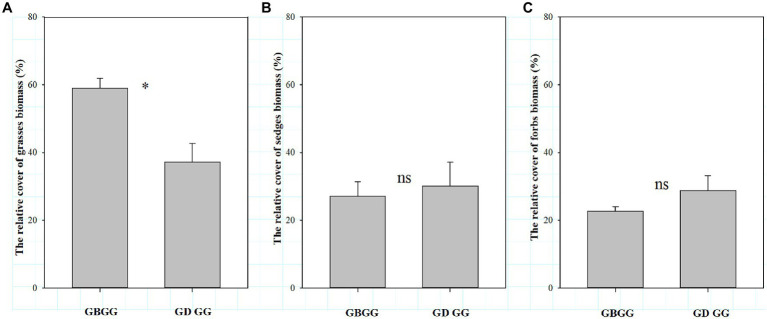
The difference in the functional group biomass % with or without grazing in grassland greenup period in winter pastures, **(A)** The relative cover of grasses biomass (%); **(B)** The relative cover of sedges biomass (%); **(C)** The relative cover of forbs biomass (%). GBGG: grazing terminated before grassland greenup; GDGG: grazing during grassland greenup. * indicates significant difference at 0.05 and ns indicates no significant difference at 0.05 levels.

**Figure 5:**
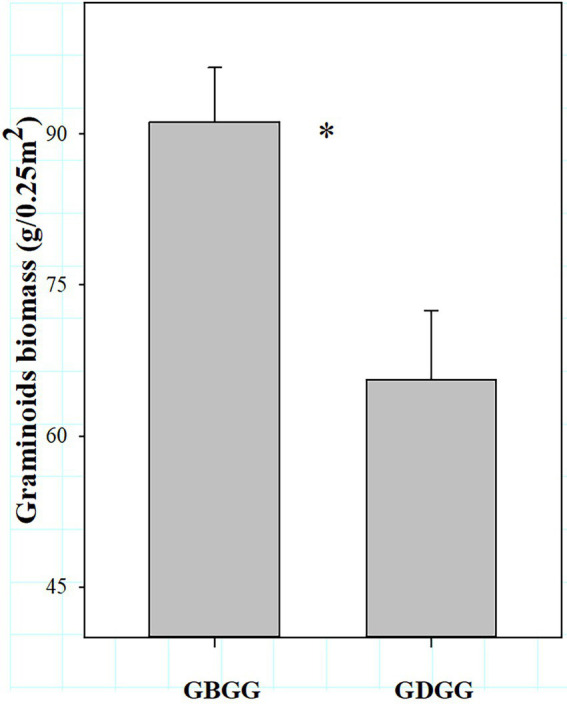
The difference in graminoid biomass (grass biomass + sedge biomass) with or without grazing in the grassland greenup period in winter pastures. GBGG, grazing terminated before grassland greenup; GDGG, grazing during grassland greenup. * indicates significance at the 0.05 level.

In winter, the changes of the three plant functional group traits are not consistent between grazing during and before grassland greenup period. The forbs richness was higher, and the grass biomass and grass biomass% were lower when grazing happened during the grassland greenup period compared to when grazing happened before the grassland greenup period (Figures 3C,D, 4A). However, the species richness of grasses and sedges, the sedge biomass and sedge biomass %, and forbs biomass and forbs biomass % were similar in winter pasture when grazing happened during the grassland greenup period compared to when grazing happened before the grassland greenup period (Figures 3A,B, 4E,F). In addition, the graminoid biomass was lower when grazing happened during the grassland greenup period compared to when grazing happened before the grassland greenup period (Figure 5).

### The potential direct and indirect influencing factors of grazing on total plant species richness and forbs richness in grassland greenup period

**Figure 6:**
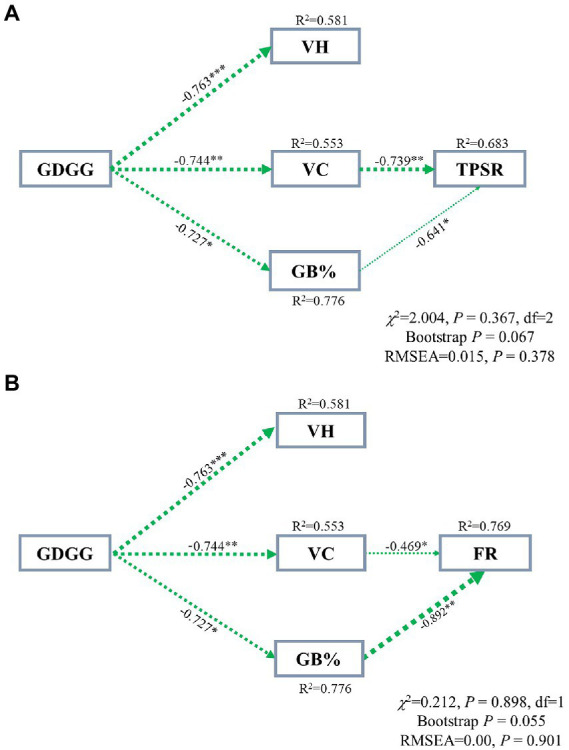
Structural equation model (SEM) exploring the direct and indirect consequences of grazing in grassland greenup period on the total plant species richness **(A)** and forbs richness **(B)** in winter pastures. Significant negative associations between any two variables are indicated by the dotted green lines, with the line width indicating the strength (average effect size) of partial regressions, while the direction of the arrow indicates probable or casual relations between two variables. The non-significant linkages were not shown in the SEM. The values adjacent to the arrows indicate the effect size of the relationship. R^2^ represent the proportion of variance explained for each dependent variable in the model. Stars indicate significant correlations. ^*^p < 0.05, ^**^p < 0.01, ^***^p < 0.001. GDGG, grazing during grassland greenup; VH, vegetation height; VC, vegetation cover; GB%, grass biomass%; TPSR, total plant species richness; FR, forbs richness.

The final piecewise SEM (standardized path coefficients are given in Supplementary Tables S2A,B), which explained 68.3% and 79.6% (R^2^ = 0.683 and 0.796) of the variance in total plant species richness and forbs richness that experienced grazing during grassland greening period, respectively (Figure 6). Results from the SEM suggested that the total plant species richness and forbs richness were higher when grazing happened during the grassland greenup period compared to when grazing was terminated before the grassland greenup period, as a result of the lower vegetation cover and grasses biomass% (Figure 6).

## Discussion

Grazing is the most important biological disturbance factor in grassland ecosystems (Li et al., 2019). The influence of grazing on vegetation community depends on the grazing intensity, grazing livestock species as well as other factors (Concostrina-Zubiri et al., 2014; Li et al., 2019). Compared to previous studies (Li et al., 2019; Freitag et al., 2021; Wilcox et al., 2022), our study is the first to analyze the difference in vegetation communities between winter pastures that were grazed during and before the grassland greenup period. Our results provide valuable information that may be used in plant diversity conservation at the scale of family pastures in alpine meadows on the QTP.

We show that vegetation height was lower when grazing happened during the greenup period compared to when grazing happened before the greenup. The main reason is that grazing during this period suppresses the growth of the tall grasses, which is the constructive species that has a decisive role for community height (Wei et al., 2019; Bricca et al., 2020). Consistent with changes in vegetation height, vegetation cover was lower when the winter pastures were grazed during the greenup period. Two reasons can be used to explain this pattern. First, the biomass of young plants is low during the greenup period, and the frequent trampling and movement of livestock (commonly known as running green) increases the soil compactness (Ma et al., 2017), which is not conducive to the rapid growth and germination of plants and the settlement of seed plants (Binks et al., 2015), and then indirectly affects the vegetation coverage. Second, the grassland greenup period is essential for the growth of plants, grazing during this period suppresses or interrupts the growth of clump grasses with more leaves (Ma et al., 2017; Zhang et al., 2020), resulting in a decrease in vegetation coverage. Similar to changes in vegetation height and cover, this study shows that the aboveground biomass was also lower when grazing happened during the greenup period. From our results it is evident that the decrease in aboveground biomass was mainly due to the decrease in grass biomass (Figure 4A). This is because the vertical height of grasses is generally higher than that of sedges and forbs, and grasses tend to occupy higher spatial niches during the greenup period (Nie and Zollinger, 2012). The intensity and likelihood of livestock disturbance on the taller plants was higher, and with greater effects on vegetative organs and tiller tissues (Benot et al., 2019). Therefore, the reduction of the biomass in grass is the result of selective feeding of fast-growing grasses by livestock.

Plant diversity provides the foundation of plant community stability and is key to maintaining the health and stability of grassland ecosystems (Tilman et al., 2006). Therefore, plant diversity can reflect the stability of grassland plant communities to a certain extent (Tilman et al., 2001), and plant species richness represents species diversity. The grasses and sedges account for less than 20% of total plant species richness in alpine meadows on the QTP (Wang et al., 2004; Li et al., 2016; Yang et al., 2020). It is evident that a change in forbs richness has a strong impact on total plant species richness, which was also clear from our study (Figures 2D, 3C). Our results show that total plant species richness was higher when grazing happened during the greenup period compared to when grazing happened before the greenup, and the higher total plant species richness resulted mainly from the higher of forbs richness, which is consistent with our first hypothesis. In addition, this study shows that grazing during the grassland greenup period is potentially beneficial to the increase of forbs richness in winter pastures, which is consistent with the second hypothesis. Previous studies found that the grassland plant richness is negatively correlated with vegetation height, cover and biomass, which limits the use of light by understory plants (Hautier et al., 2009; Borer et al., 2014; Li et al., 2016). The results of SEM potentially showed that grazing during the grassland greenup period affected total plant species richness and forbs richness indirectly by reducing vegetation cover and the grass biomass % (Figures 6A,B). This confirms that grazing removes some tall grasses on the ground, reduces the canopy density of the vegetation community (Wei et al., 2019), and the competition for light resources (Hautier et al., 2009; Borer et al., 2014), and allows the forbs that are disadvantaged by competition to survive. In addition, the increase in forbs richness may also be due to the frequent trampling and movement of livestock, which creates some bare ground and can provide space for some annual and grazing-tolerant forbs species to settle (Nie and Zollinger, 2012). However, the changes in total plant species richness and forbs richness were not caused by the changes in vegetation height. Although the vegetation height was lower when grazing happened during the greenup period compared to when grazing happened before the greenup, its height still reached up to 12.9 cm. Thus vegetation height is not the decisive factor that affects total plant species richness under the background of grazing during the greening period. Indeed, grazing livestock species and soil physicochemical properties can also influence the plant species richness (Nie and Zollinger, 2012). However, we did not consider these influencing factors because plant richness is primarily controlled by light, regardless of soil nutrients and livestock utilization (Hautier et al., 2009; Borer et al., 2014).

Generally, according to the theory of niche complementarity (Tilman et al., 2006), if the biomass of one or several functional groups in the community decreases, it will inevitably lead to the increase of the biomass of other functional groups. However, the decrease in grass biomass did not lead to an increase in sedges or forbs biomass (Figures 3B,C). There were no obvious changes in sedge biomass, and it may be related to the growth characteristics of sedges. The livestock feeding during the grassland greenup period can inhibit the upward growth of sedges (Selemani et al., 2013), but it will simultaneously increase their clonal reproduction (Binks et al., 2015), therefore there is no change in sedge biomass. Forb biomass was similar when grazing happened during the greenup period compared to when grazing happened before the greenup, potentially because most forbs are dwarf and creeping plants which has a limited competitive ability and living space (David et al., 2018, 2020). Whether plants can grow normally in natural grasslands is affected by both their own botanical characteristics and environmental factors (Binks et al., 2015), and by livestock feeding. A reasonable resting period from grazing can prevent plants from being disturbed, create a good space for their growth and reproduction, and is conducive to the positive succession of plant communities (Dong et al., 2020). In addition, a resting period from grazing during the grassland greenup period is conducive to the accumulation of aboveground biomass, increasing the photosynthetic capacity of the community vegetation, and then feeding back to the aboveground biomass (Ma et al., 2017). Therefore, the results of this study confirm that grazing prohibition during the greening period of grassland is beneficial to the restoration of degraded grassland vegetation.

## Conclusion

We analyzed the differences in vegetation characteristics between grazing winter pastures where grazing was terminated before and during the grassland greenup period. We found that the total plant species richness was higher in grassland with grazing during the grassland greenup period and was mainly the result of the higher forbs richness compared to when grazing was terminated before the greenup period. Our results imply that grazing during the grassland greening period maybe is facilitating for the plant diversity conservation of winter pastures in alpine meadow at the family pasture scale from the perspective of sustainable development of grassland ecosystems. In addition, this results also imply that the prohibition of grazing during the grassland greenup period may facilitate to the improvement of grassland productivity in winter pasture of alpine meadow at the family pasture scale.

## Data availability statement

The raw data supporting the conclusions of this article will be made available by the authors, without undue reservation.

## Author contributions

WW designed the study and wrote the first draft of the manuscript. WW, QZ, and MO analyzed the data. WW, HY, JD, MQ, and QZ conducted fieldwork. All authors contributed to the article and approved the submitted version.

## Funding

This study was supported by the Fundamental Research Funds of China West Normal University (18Q046) and the Gansu Provincial Science and Technology Program (1054nkcp159).

## Conflict of interest

The authors declare that the research was conducted in the absence of any commercial or financial relationships that could be construed as a potential conflict of interest.

## Publisher’s note

All claims expressed in this article are solely those of the authors and do not necessarily represent those of their affiliated organizations, or those of the publisher, the editors and the reviewers. Any product that may be evaluated in this article, or claim that may be made by its manufacturer, is not guaranteed or endorsed by the publisher.
