# Supplementary material for: Grazing during the grassland greenup period promotes plant species richness in alpine grassland in winter pastures
Source: Front Plant Sci. 2022 Aug 16;13:973662. doi: 10.3389/fpls.2022.973662 (PMC9426632; doi:10.3389/fpls.2022.973662)

**Supporting Information**

**This file includes:**

Table S1

Figure S1

**Table S1.** Spearman’s correlation between vegetation height (VH), vegetation cover (VC) and the grass functional biomass% (GB%).

| Variables | VH | VC | GB% |
| --- | --- | --- | --- |
| VH | 1 |  |  |
| VC | 0.540 | 1 |  |
| GB% | 0.593 | 0.329 | 1 |

**Table S2.** Coefficient estimates from the final piecewise structural equation model relating how grazing during grassland greenup period (GDGG) affects the total plant species richness (a, TPSR) and the forbs richness (b, FR) via plausible pathways include the vegetation cover, vegetation height and grass biomass% (GB%). Shown are the standardized path coefficients (scaled by their mean and standard deviation), standard error of coefficient, and the levels of significance for corresponding pathways (*P* value). Significant pathways (*P* < 0.05) are in bold.

|  | **Response** | **Predictor** | **Standard coefficient** | **Standard error** | ***P*** |
| --- | --- | --- | --- | --- | --- |
| a | VC | GDGG | **-2.05** | 0.652 | **0.02** |
|  | VH | GDGG | **-1.487** | 0.446 | **< 0.001** |
|  | GP% | GDGG | **-0.106** | 0.046 | **0.022** |
|  | GP% | VC | -0.025 | 0.013 | 0.054 |
|  | GP% | VH | 0.038 | 0.019 | 0.051 |
|  | TPSR | VH | **1.052** | 0.927 | 0.257 |
|  | TPSR | GP% | **-21.337** | 10.902 | **0.049** |
|  | TPSR | VC | **-1.299** | 0.433 | **0.003** |
| b | VC | GDGG | **-2.05** | 0.652 | **0.02** |
|  | VH | GDGG | **-1.488** | 0.446 | **< 0.001** |
|  | GP% | GDGG | **-0.106** | 0.046 | **0.022** |
|  | GP% | VC | -0.025 | 0.013 | 0.054 |
|  | GP% | VH | 0.038 | 0.019 | 0.051 |
|  | FR | VH | **0.822** | 0.781 | 0.293 |
|  | FR | GP% | **-29.332** | 9.189 | **< 0.01** |
|  | FR | VC | **-0.815** | 0.365 | **0.026** |

**Figure S1.** Hypothesized effects of the grazing during the grassland greenup period (GDGG) on the total plant species richness (a, TPSR) and forbs richness (b, FR) by affecting vegetation height (VH), vegetation cover (VC) and grass biomass% (GB%).


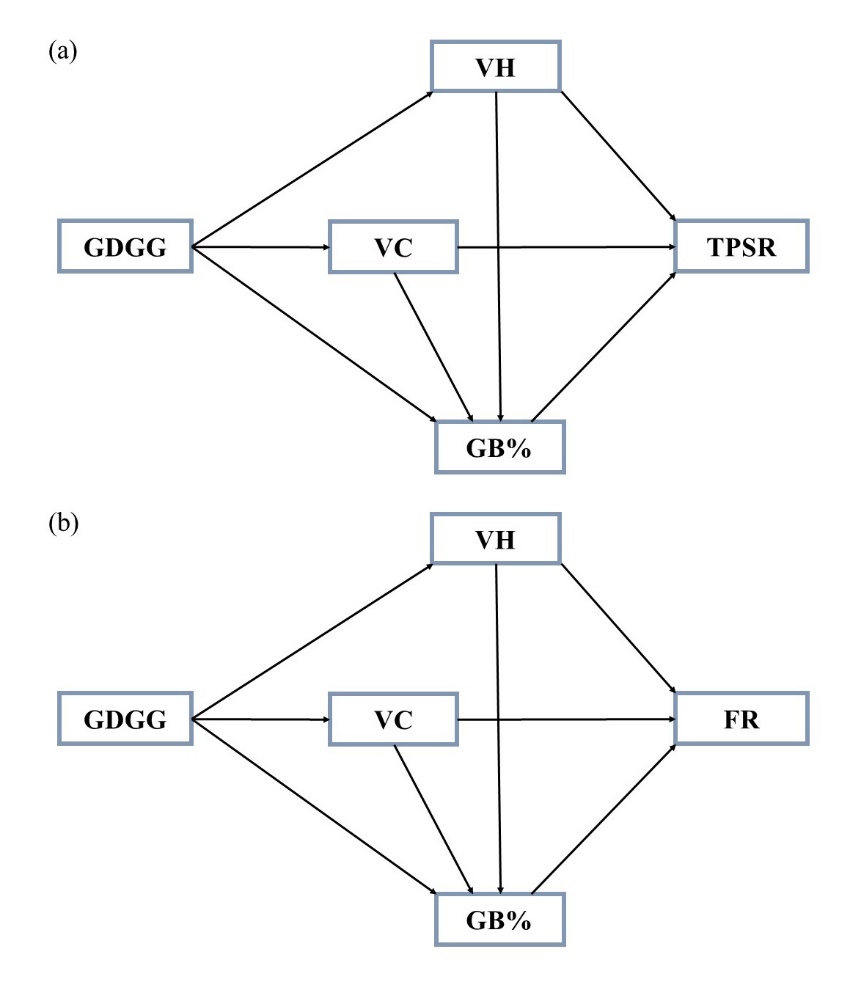

Supplement: Supplementary file 1 [file Data_Sheet_1.docx]
